# A kinesin motor in a force-producing conformation

**DOI:** 10.1186/1472-6807-10-19

**Published:** 2010-07-05

**Authors:** Elisabeth Heuston, C Eric Bronner, F Jon Kull, Sharyn A Endow

**Affiliations:** 1 Department of Chemistry, Dartmouth College, Hanover, NH 03755 USA; 2 Department of Cell Biology, Duke University, Durham, NC 27710 USA

## Abstract

**Background:**

Kinesin motors hydrolyze ATP to produce force and move along microtubules, converting chemical energy into work by a mechanism that is only poorly understood. Key transitions and intermediate states in the process are still structurally uncharacterized, and remain outstanding questions in the field. Perturbing the motor by introducing point mutations could stabilize transitional or unstable states, providing critical information about these rarer states.

**Results:**

Here we show that mutation of a single residue in the kinesin-14 Ncd causes the motor to release ADP and hydrolyze ATP faster than wild type, but move more slowly along microtubules in gliding assays, uncoupling nucleotide hydrolysis from force generation. A crystal structure of the motor shows a large rotation of the stalk, a conformation representing a force-producing stroke of Ncd. Three C-terminal residues of Ncd, visible for the first time, interact with the central β-sheet and dock onto the motor core, forming a structure resembling the kinesin-1 neck linker, which has been proposed to be the primary force-generating mechanical element of kinesin-1.

**Conclusions:**

Force generation by minus-end Ncd involves docking of the C-terminus, which forms a structure resembling the kinesin-1 neck linker. The mechanism by which the plus- and minus-end motors produce force to move to opposite ends of the microtubule appears to involve the same conformational changes, but distinct structural linkers. Unstable ADP binding may destabilize the motor-ADP state, triggering Ncd stalk rotation and C-terminus docking, producing a working stroke of the motor.

## Background

Motor proteins of the kinesin family hydrolyze ATP and use the energy released by nucleotide hydrolysis to move along microtubules, performing essential roles in transport, division and other cellular processes. The mechanism by which motors produce force to move on microtubules is not fully understood and remains an outstanding problem in the field. A prevailing hypothesis is that the motor undergoes a conformational change that, under load, produces strain. The strain is relieved by a force-producing movement that displaces the motor relative to the microtubule [1]. Coupling of steps of ATP hydrolysis to the force-producing structural changes of the motor is thought to drive motor movement along microtubules.

Progress in understanding the motor mechanism has come from the discovery of the kinesin-14 motors. The motors in this kinesin group bind to microtubules and move towards the more stable, slow polymerizing and depolymerizing minus ends, the opposite direction as the first discovered kinesin, kinesin-1. The kinesin-14 motors include Ncd, a motor that plays an essential role in spindle assembly in *Drosophila *oocytes and functions in the spindle and at the poles in early embryos. Structural studies revealed that Ncd differs from kinesin-1 in that the conserved motor domain or head is joined directly to the α-helical coiled-coil stalk, rather than containing a 'neck linker' between the head and stalk [2,3]. The kinesin-1 neck linker consists of two β-strands that dock onto and undock from the motor core, thereby allowing each head of the dimeric motor to reach the next binding site along a microtubule. This permits the motor to move processively and take many successive steps each time it binds to a microtubule [4-6].

The tight coupling between ATP hydrolysis and kinesin-1 steps [7,8] means that changes in nucleotide binding or hydrolysis by the motor can greatly affect motor stepping. An example is kinesin-1 T94S, which is mutated for a residue in the nucleotide-binding GQ**T**SSGKT motif or P-loop - the change of an invariant threonine to a serine causes the motor to release ADP faster than wild type and to take successive 16-nm steps under high load, instead of 8-nm steps like wild-type kinesin-1 [9]. The 16-nm steps consist of rapid double 8-nm steps with a short dwell between steps, followed by a longer dwell. The mutation affects a step in the hydrolysis cycle that is sensitive to load and the nucleotide state of the motor, producing alternating long and short dwells that alter kinesin-1 stepping along microtubules. Load-sensitive steps of the cycle are thought to correspond to force-producing steps of the motor, which have not yet been identified with certainty for kinesin-1. These findings raise the possibility that ADP release is a force-producing step of the kinesin cycle.

Ncd, by contrast, is a nonprocessive motor that undergoes a single displacement along a microtubule, then releases from the microtubule [10,11], although the motor has been reported to move processively under certain *in vitro *conditions [12]. The force-producing stroke of Ncd is thought to consist of a large rotation of the coiled-coil stalk [10,13] that occurs when the motor binds to a microtubule and releases ADP [10], or, alternatively, after ADP release, when the microtubule-bound motor binds ATP [11,14,15].

The effects of an uncoupling mutation like kinesin-1 T94S on a nonprocessive kinesin motor are not known, given that little is known about force generation by the motors. One possibility is that the mutant might reveal a phase important for force production, which could differ from kinesin-1, giving unexpected insights into the motor mechanism of function. Here we analyze Ncd, a nonprocessive motor, with the corresponding mutation, using kinetic and structural methods. We find that NcdT436S releases ADP and hydrolyzes ATP faster than wild type, like kinesin-1 T94S. Unexpectedly, an NcdT436S crystal structure shows a large rotation of the α-helical coiled-coil stalk, resembling a previous structure of an Ncd motor with a mutation in the microtubule-binding site, NcdN600K [13]. Three more residues of the C-terminus are visible for the first time in the NcdT436S model. Remarkably, the residues dock onto the central β-sheet in one head of the dimeric motor in a conformation similar to the neck linker of kinesin-1 and other plus-end motors, resembling the kinesin-14 KCBP 'neck mimic' [16]. The unstable binding to ADP by NcdT436S, together with its crystal structure, imply that rotation of the Ncd stalk occurs with ADP release and docking of the C-terminus onto the motor core, producing force. ADP release by the Ncd motor could destabilize the motor-ADP state and cause a head to undock from its neck, triggering the stalk rotation and C-terminus docking to produce a working stroke of the motor.

## Results

### NcdT436S

**Figure 1:**
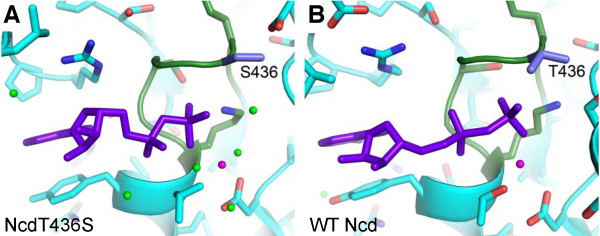
**NcdT436S**. *A*, T436S (blue) lies in the P-loop (green). ADP, purple; Mg^2+^, magenta sphere; water, green spheres. See also Additional file 1 Figure S1. *B*, Wild-type Ncd (PDB 1CZ7) [17] with T436 (blue).

NcdT436S was designed to obtain information about the force-generating step of a nonprocessive kinesin motor. The mutation changes an invariant threonine in the nucleotide-binding motif, GQ**T**GSGKT, to a serine (Figure 1). The threonine-to-serine mutation is predicted to open the nucleotide-binding cleft and make the P-loop more closely resemble that of the myosins, GE**S**GAGKT; this is expected to permit more rapid nucleotide exchange, potentially enabling the Ncd motor to be crystallized in a different state than previous motor-ADP structures [2,17]. NcdT436S was expressed in bacteria as an N-terminal glutathione S-transferase (GST) fusion protein for biochemical and motility assays, or as a nonfusion protein for structural analysis by X-ray crystallography.

### NcdT436S nucleotide release and hydrolysis

**Figure 2:**
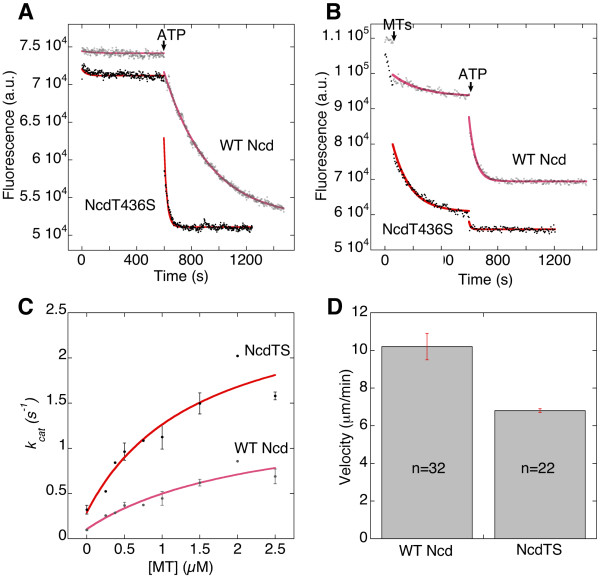
**NcdT436S nucleotide hydrolysis kinetics and motility**. *A*, Mant-ADP release upon adding ATP (arrow). Assays performed in HEM100. NcdT436S (black, red curve fit); wild type (WT) (grey, pink curve fit). a.u., arbitrary units. *B*, Mant-ADP release upon adding excess microtubules (MTs, arrow), then ATP (arrow). Assays performed in HEM50. NcdT436S (black, red curve fit) released ADP with no additions, then, after adding microtubules, released most of the remaining bound ADP; the rest was released by adding ATP. By contrast, wild type (grey, pink curve fit) released about half the bound ADP with microtubules and the rest upon adding ATP. *C*, Basal and microtubule-stimulated ATPase activity in steady-state assays. NcdT436S (black, red curve fit); wild type (grey, pink curve fit). k_cat _± sem. *D*, In vitro motility. Leading-end microtubule gliding velocity ± sem on NcdT436S and wild type.

The NcdT436S mutation lies in the nucleotide-binding cleft and is expected to alter ADP binding by the motor; we therefore measured the ADP release rate by the NcdT436S mutant when excess ATP was added, using a fluorescent ADP analogue, mant-ADP, bound to the motor. The assays, performed in HEM100 (100 mM NaCl), showed a 10-fold higher rate constant for NcdT436S (k_off _= 0.0304 ± 0.0005 s^-1^, n = 6) than wild-type Ncd (k_off _= 0.00305 ± 0.00002 s^-1^, n = 6) (Figure 2A and Table 1), indicating much faster ADP release, consistent with weak ADP binding. Assays in lower salt (HEM50, 50 mM NaCl), like that used in a previous study of Ncd [18], showed significant ADP release from NcdT436S with no additions (26 ± 8%, n = 4; k_off _= 0.0035 ± 0.0005 s^-1^, n = 2); after adding 4-5 fold excess microtubules, almost all the remaining bound ADP was released (69 ± 8%, k_off _= 0.014 ± 0.003 s^-1^, n = 4) and the rest was released by adding ATP (4 ± 2%, n = 4; k_off _= 0.066 ± 0.009 s^-1^, n = 3) (Figure 2B and Table 1). By contrast, wild-type Ncd released a small amount of ADP with no additions (6 ± 3%, n = 6; k_off _= 0.00003 ± 0.00001 s^-1^, n = 2), approximately half the bound ADP upon addition of 4-5 fold excess microtubules (47 ± 5%, k_off _= 0.018 ± 0.006 s^-1^, n = 6) and the remaining half upon adding ATP (47 ± 8%, k_off _= 0.04 ± 0.01 s^-1^, n = 6), similar to findings by others [18]. The mant-ADP assays with microtubules show that ADP release occurs in two steps in NcdT436S, rather than a single step as reported for monomeric NcKin3 [19]. The increased size of the first step compared to wild type indicates that ADP in both heads of NcdT436S is more weakly bound than wild type.

**Table 1:** NcdT436S kinetics and motility

Motor	ADP Release* k_off _(s^-1^)			ATPase			Velocity (μm/min)	
	
	No MTs + ATP	+ MTs	+ MTs, ATP	No MTs k_cat _(s^-1^)	+ MTs k_cat _(s^-1^)	K_m,MTs_ (μM)	Leading end	Lagging end
NcdT436S	0.0304 ± 0.0005 (n = 6)	0.014 ± 0.003 (n = 4)	0.066 ± 0.009 (n = 3)	0.28 ± 0.10 (n = 4)	2.2 ± 0.4 (n = 4)	1.3 ± 0.6 (n = 4)	6.8 ± 0.1 (n = 22)	7.6 ± 0.2 (n = 22)

WT Ncd	0.00305 ± 0.00002 (n = 6)	0.018 ± 0.006 (n = 6)	0.04 ± 0.01 (n = 6)	0.10 ± 0.04 (n = 4)	1.0 ± 0.2 (n = 4)	1.6 ± 0.8 (n = 4)	10.2 ± 0.7 (n = 32)	10.9 ± 0.7 (n = 31)

MTs, microtubules. Mean ± sem.

*No MTs assays in HEM100; + MTs assays in HEM50

Weak ADP binding could alter the motor ATP turnover rate by overcoming the rate-limiting step, ADP release [20]. The NcdT436S ATP hydrolysis rate was measured in steady-state ATPase assays (Figure 2C and Table 1). The basal rate without microtubules (0.28 ± 0.10 s^-1^, n = 4) was 2.8-fold faster than wild type (0.10 ± 0.04 s^-1^, n = 4). With microtubules, the NcdT436S mutant (2.2 ± 0.4 s^-1^, n = 4) hydrolyzed ATP ~2-fold faster than wild type (1.0 ± 0.2 s^-1^, n = 4), after correcting for hydrolysis by microtubules. The NcdT436S K_m,MTs _(1.3 ± 0.6 μM, n = 4) overlapped with wild type (1.6 ± 0.8 μM, n = 4), indicating that the mutant binding affinity for microtubules does not differ significantly from wild type. Thus, NcdT436S hydrolyzes ATP faster than wild type in the presence or absence of microtubules; the faster ATPase of the mutant can be attributed to faster ADP release than wild type, rather than a change in microtubule-binding affinity. These results are consistent with the interpretation that the NcdT436S mutant binds more weakly to ADP, destabilizing the motor-ADP state, the most stable state of the kinesin motors [20].

### NcdT436S microtubule gliding assays

Faster microtubule-stimulated ATP hydrolysis than wild type is predicted to result in faster microtubule gliding, if force generation by the motor is tightly coupled to ATP hydrolysis, as observed for kinesin-1 [7,8]. Motility assays were performed to measure the velocity of microtubules gliding on ensembles of motors attached to a coverslip surface. Unexpectedly, the NcdT436S gliding velocity was slower than wild type. We also observed significantly faster lagging-end (7.6 ± 0.2 μm/min, n = 22) than leading-end velocity (6.8 ± 0.1 μm/min, n = 22) for microtubules gliding on NcdT436S, but not on wild-type Ncd (lagging end, 10.9 ± 0.7 μm/min, n = 31; leading end, 10.2 ± 0.7 μm/min, n = 32) (Table 1). Microtubules shortened as they glided on NcdT436S, consistent with disassembly at the minus ends, which would produce faster lagging- or minus-end velocity [21]. The leading-end velocity was taken to be the actual gliding velocity - it was 1.5 times slower for NcdT436S than wild type (Figure 2D). The slower gliding velocity but faster rate of ATP hydrolysis indicates that NcdT436S movement along microtubules is uncoupled from ATP hydrolysis. The motor may undergo futile ATP hydrolyses in which the energy of hydrolysis is dissipated rather than converted into force. The T436S mutation thus increases the rate of ADP release, destabilizing the motor-ADP state, but uncouples ATP hydrolysis from force generation and motor movement along microtubules.

### NcdT436S crystal structure

**Figure 3:**
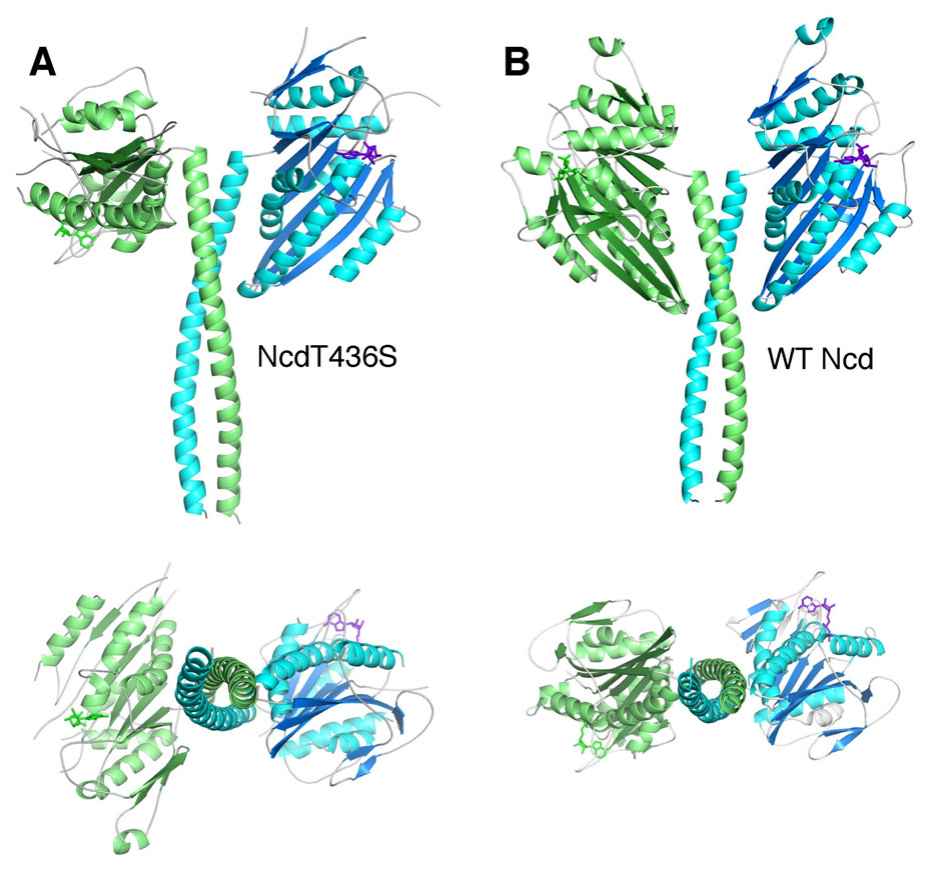
**NcdT436S crystal structure**. *A*, NcdT436S heads are oriented asymmetrically relative to the coiled-coil stalk. Chain A, cyan; chain B, green. *B*, Wild-type Ncd (PDB 1CZ7) [17] heads show two-fold symmetry around the axis of the stalk. Bottom, structures rotated 90°; the stalk extends out of the plane of the page highlighting the different orientation of the chain B head.

**Figure 4:**
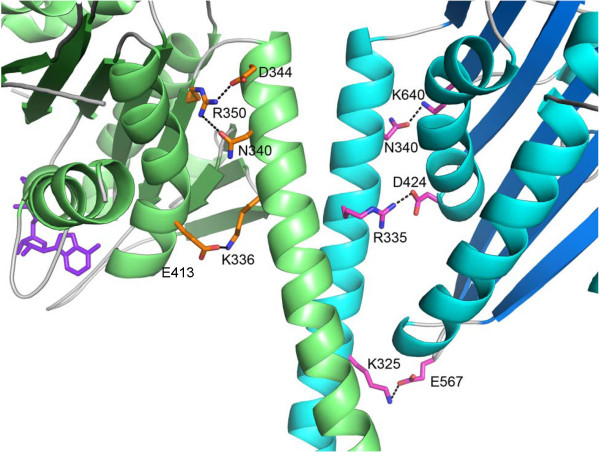
**Salt bridges between the motor core and neck in NcdT436S**.Distinct salt bridges between the neck and motor core of chain A (cyan) and B (green) stabilize the orientation of the heads with respect to the neck, as indicated. Dimer orientation the same as Figure 3A (top).

The weak binding by NcdT436S to ADP suggested that crystallization might reveal a different conformation than the previously reported motor-ADP state [2,17]. NcdT436S was crystallized and the structure was solved to 2.8 Å (Table 2). Remarkably, the model showed a large rotation of the α-helical coiled-coil stalk together with one of the two heads, resulting in asymmetry of the heads (Figure 3), resembling a previously reported crystal structure of NcdN600K [13]. The NcdT436S stalk is rotated by ~70° when the head of chain B, which does not rotate with the stalk, is aligned with one of the two heads of a wild-type Ncd structure (PDB 1CZ7) [17]. Salt bridges between the stalk and motor core that differ between the two chains stabilize the stalk: N340-K640, R335-D424 and K325-E567 in chain A and D344-R350, N340-R350 and K336-E413 in Chain B (Figure 4). The chain B head shows disrupted interactions with neck residues that are observed in chain A and is positioned to interact with the microtubule, resembling NcdN600K head H2, whereas the chain A head interacts extensively with residues of the neck and has rotated with the stalk, like NcdN600K head H1. The resemblance between the NcdT436S and NcdN600K structures is unexpected, given that the residue mutated in NcdT436S is in the nucleotide-binding cleft instead of the microtubule-binding site, as in NcdN600K. Superposition of the models shows that the overall conformations of the two structures are highly similar, with a root mean square deviation (RMSD) of 0.905 Å over 657 alpha carbons.

**Table 2:** Crystallographic data and refinement statistics

X-ray diffraction data	
Space group	C2
Unit Cell Dimensions: *a, b, c *(Å) *α, β, γ *(°)	162.0, 66.4, 93.6 90, 98.2, 90
Wavelength (Å)	1.0088
Resolution Range (Å)	19.34 - 2.75 (2.90 - 2.75)*
R_*sym *_(%)	7.0 (36.2)
I/σ	19.92 (4.42)
Measured reflections	64394 (9470)
Unique reflections	22267 (3362)
Redundancy	2.89 (2.82)
Completeness (%)	86.0 (88.8)
Refinement	
Resolution (Å)	19.34 - 2.75
R_*cryst*_/R_*free *_(%)	23.8/29.2
Atoms (protein/ligand/solvent)	5469/56/243
Rmsd bond length (Å)	0.012
Rmsd bond angles (°)	1.6
Average B-factors (Å^2^, main chain/side chain)	66.4

* Data in parentheses represent the highest resolution shell. Deposited under PDB 3L1C.

### NcdT436S nucleotide-binding site

The ATP-binding pocket was examined for structural changes that could explain the uncoupling of ATP hydrolysis from motility by the NcdT436S motor. T436 is in the nucleotide-binding P-loop, where it forms a small cap over the cleft and interacts with the β-phosphate of ADP (Figure 1). The nucleotide-binding cleft is slightly more open in the NcdT436S structure because of the substitution of threonine by the smaller serine. Comparison of the P-loop and flanking residues (I430-T443) of NcdT436S with NcdN600K (PDB 1N6M) [13] and wild-type Ncd (PDB 2NCD, PDB 1CZ7) [2,17] shows that the NcdT436S chains superimpose with the other two models. Although the orientation of S436 could differ from T436 in the other structures, the S436 hydroxyl is in the same position as the T436 hydroxyl in previous structures and the bound ADP is also similarly positioned as in the other structures (Figure 1). Despite the overall 2.8 Å resolution of the NcdT436S structure, ordered water molecules are visible in the nucleotide-binding cleft of both heads. There is a difference in the position of at least one water molecule in the NcdT436S cleft that may explain the more rapid release of ADP by the mutant than wild type. The water molecule forms hydrogen bonds to the S436 hydroxyl group, ADP β-phosphate and G583 amide group, and is present in the head of chain A which rotates with the stalk, but is not visible in the head of chain B. Although density corresponding to the bound ADP and Mg^+2 ^is visible in both NcdT436S heads, the density for the PO_4_^-2 ^and Mg^+2 ^groups is stronger in the head of chain A than chain B (Additional file 1 Figure S1). Moreover, many of the ordered water molecules near the ADP in chain A are not ordered in chain B.

The kinesin motors contain invariant switch I and II motifs with structural homology to those in G proteins that move upon nucleotide hydrolysis and exchange. The switch I and II residues form a salt bridge in some motor-ADP structures, including wild-type Ncd (PDB 2NCD) [2]. Formation of the salt bridge in myosin closes the nucleotide-binding pocket, which is thought to enable the motor to hydrolyze ATP [22]. The salt bridge is not formed in either head of the NcdT436S or NcdN600K crystal structure; instead of forming a salt bridge with R552 of switch I, E585 of switch II interacts with S436 or T436, respectively, in the two motors, resulting in the open conformation of the NcdT436S nucleotide-binding cleft.

### NcdT436S microtubule-binding region

Two regions within the conserved motor domain (G347-K674) with large differences, 4-10 Å, between the between the two NcdT436S chains were identified at the N terminus of loop L12 (Q614-Q616) and the C-terminus of the visible structure (residues C670-K674).

Loop L12, together with the adjacent loop L11 and flanking helices α4 and α5, forms the microtubule-binding interface of the kinesin motors. The two NcdT436S chains differ in the length and conformation of loop L12, as well as helices α4 and α5. Loop L12 is shorter and helix α4 is longer in the head of chain A that rotates with the stalk, compared to the head of chain B. Helix α5 is longer by one residue in chain A and forms a kink that is not present in chain B. This difference in helix α5 between the two heads is also observed in the corresponding heads of NcdN600K [13]. However, the two structures differ in that loop L12 is visible in NcdN600K only in head H1, but not in head H2. Helix α4 of NcdN600K also differs in length between heads H1 and H2, but the helix in both heads of NcdN600K is longer than in the corresponding NcdT436S heads. Differences in the length and conformation of helix α4 and the position of loop L11-helix α5-loop L12 were observed in kinesin-3 KIF1A crystal structures and attributed to differences between the ADP and ATP state [23]. More dramatic differences have been observed in 3D cryoEM reconstructions of the kinesin-14 Kar3 bound to microtubules [24] and are thought to reflect nucleotide-specific motor conformational changes that are stabilized by interactions with microtubules.

### C-terminus docking

The two NcdT436S chains differ in their degree of order - overall, chain A, which is visible from M292 to K671, is more ordered than chain B, which is visible from G290 to K674. Chain A ends four residues after helix α6, the last structural element of the conserved motor core, whereas chain B extends three residues further. The three C-terminal residues visible in chain B have not been seen in other Ncd structures, which are disordered after helix α6 - the NcdN600K chains end at K671 and N668, and those of wild-type Ncd at M672 (PDB 2NCD) [2] or K671 (PDB 1CZ7) [17].

**Figure 5:**
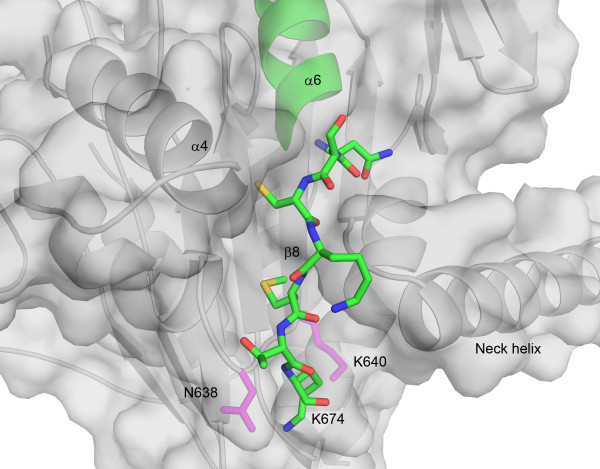
**Docking of the NcdT436S C-terminus onto the motor domain**. The C-terminal seven visible residues (NSCKMTK) after helix α6 (green) of chain B pack into a groove formed by the C-terminus of helix α4, N terminus of strand β8 of the central β-sheet, and N-terminus of the neck helix. The C-terminus of chain A (see Figure 6A) projects away from the motor domain and quickly becomes disordered.

Although only three more residues of chain B are visible in NcdT436S compared to previous Ncd crystal structures, they reveal a major difference between the two chains at their C termini. The chains show an abrupt change in orientation at A665 and chain B tilts away from chain A towards the central β-sheet. The last visible residue of chain B, K674, interacts with N638 at the N terminus of strand β8 of the central β-sheet (Figure 5). This lysine also packs near K640, a residue that, together with N340 of the neck, is required for Ncd minus-end directed motility [10]. Interactions of K674 with N638 and K640 could destabilize the N340-K640 interaction in chain B, disrupting interactions of the head with the neck, and allow the stalk to rotate with the chain A head in which the N340-K640 interaction is maintained.

**Figure 6:**
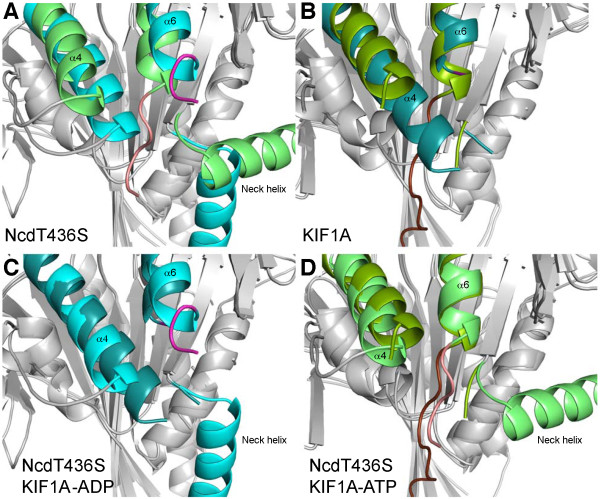
**NcdT436S vs KIF1A**. *A*, NcdT436S chain A (cyan) and B (green) differ in C-terminus orientation (chain A, magenta; chain B, pink), helix α4 differs in length and the neck helices show distinct trajectories as they leave the motor domain to form the coiled-coil stalk. *B*, Helix α4 is longer by two turns in KIF1A-ADP (dark cyan) than KIF1A-AMP·PNP (dark green; C-terminus, brown). *C*, Helix α6 is oriented similarly in NcdT436S chain A and KIF1A-ADP. *D*, The C-terminus of NcdT436S chain B and KIF1A-ATP (KIF1A-AMP·PNP) are similarly oriented and helix α4 is similar in length. See also Additional file 1 Figure S2.

Its movement onto the motor core inserts the C-terminus of chain B between loop L12 and the end of the neck helix, G347-N348. This may displace loop L12 from its position at the end of the neck and cause the small (1.5 Å) translation in helix α4 along the helix axis observed in chain B (Figure 6). The changes in loop L12 may reflect nucleotide-dependent changes that may be stabilized by motor interactions with microtubules, as noted above. The NcdT436S structure shows the C-terminal residues of chain B, which contains the head positioned to interact with the microtubule, docked onto the motor core, but the head undocked from the neck. In kinesin-1 and other plus-end directed motors, the neck linker adjacent to the conserved motor core attaches the catalytic domain to the coiled-coil stalk. The NcdT436S structure shows that the Ncd C-terminal residues are in the same position as the neck linker of plus-end kinesins, resembling the C-terminal 'neck mimic' of kinesin-14 KCBP [16]. Remarkably, both the position of the docked C-terminus and the orientation of helix α4 in NcdT436S chain B are similar to the docked neck linker and helix α4 of a kinesin-3 motor, KIF1A, in the ATP (AMP-PNP) state (Figure 6). Furthermore, the disorder of C-terminal residues beyond helix α6 in chain A and the orientation of helix α4 are similar to that seen in KIF1A in the ADP state.

## Discussion

### Kinesin force generation - the neck linker

The mechanism by which the kinesin motors produce force to move along microtubules or disassemble microtubules is still not well understood. In kinesin-1 and other plus-end kinesins, the neck linker, consisting of two short β-strands separated by a loop, attaches the N-terminal catalytic domain to the α-helical coiled-coil stalk; the neck linker is observed either docked onto or undocked from the motor core in crystal structures. Kinetic and structural studies have led to a model in which nucleotide-induced conformational changes cause the neck linker to dock onto the motor core in the ATP state and undock in other nucleotide states, producing force that drives motility [25]. However, the proposal that neck linker docking is the primary force-producing mechanical event of the plus-end kinesins has been controversial because of the relatively small free energy change associated with its docking [26].

Recently, a β-strand at the N-terminus of the motor - the 'cover strand' - has been hypothesized to interact with the neck linker in the ATP state, forming a β-sheet or 'cover-neck bundle' that helps dock the neck linker onto the motor core, producing forward motion [27,28] (Additional file 1 Figure S2). Molecular dynamics simulations showed that the cover-neck bundle can generate greater force than the neck linker alone and that the neck linker without the cover strand is flexible and cannot produce sufficient force for forward movement by the motor [27]. Motors with a deletion of the cover strand or point mutations in the cover strand that would hinder cover-neck bundle formation showed impaired motility, providing functional evidence for the requirement of the cover strand and cover-neck bundle in kinesin motiity [28].

It has been postulated that the Ncd C-terminus might play a role similar to the kinesin-1 cover strand in force generation [27,28]. The NcdT436S structure confirms this - in kinesin-1 and other plus-end kinesins, the cover strand at the motor N-terminus is proposed to bind to the motor core in the ATP state together with the neck linker; in Ncd, C-terminal residues corresponding to the cover strand bind directly to the motor core forming a structure that resembles the neck linker of the plus-end kinesins. The major difference between Ncd and plus-end kinesins is that docking of the C-terminal residues does not require a separate cover strand - the docked structure resembles the kinesin neck linker in its conformation and also functions as a cover strand over the binding cleft. In this case, the salt bridges between the docked C-terminus and motor core could compensate for the lack of a cover strand. It is also possible that subsequent residues of the Ncd C-terminus, which contains 26 more residues following K674, fold back to form a cover strand. Although the thermodynamics of the docked state are difficult to predict from the residues involved and distances alone, the breaking of salt bridges present in the undocked state and formation of new salt bridges in the docked state represents a bistable switch that can stabilize the stalk-rotated conformation of the motor.

A crystal structure of kinesin-14 KCBP, thought to represent the ATP state, shows the C-terminus docked onto the motor core, forming the 'neck mimic', resembling the kinesin-1 neck linker [16]. The KCBP C-terminus performs a different role than the C-terminus of other kinesin-14 motors in regulating Ca^+2-^calmodulin binding by the motor, which undocks the neck mimic and inhibits motor binding to microtubules in the ADP state [29]. The finding that the Ncd C-terminus resembles the KCBP neck mimic is unexpected, given that there is no apparent inhibitory binding partner for Ncd. Nonetheless, Ncd binding to microtubules is weak in the motor-ADP state in which docking of the C-terminus is sterically blocked by extensive interactions of the head with the neck at the end of the stalk; rotation of the stalk away from one head to produce a power stroke allows the C-terminus to dock onto the motor core and the motor to bind to microtubules. The Ncd C-terminus could thus regulate microtubule binding without an inhibitory binding protein, representing a different mechanism of regulation than KCBP.

The requirement for the Ncd C-terminus in motor function has been demonstrated in motility assays of chimeric Ncd-kinesin-1 motors - a chimera that lacked the Ncd C-terminus did not bind microtubules to the glass surface [30]. The Ncd C-terminus is 36 residues long and contains a relatively high number of positively charged amino acids (4 lysines + 2 arginines) which, in addition to interacting with the motor core, could mediate motor binding to microtubules by charge-charge interactions. Strikingly, addition of the Ncd C-terminus to the chimera resulted in motility, either plus- or minus-end directed, depending on the neck-motor junction. Moreover, replacing the C-terminus of wild-type Ncd with the kinesin-1 neck linker produced slow minus-end movement [30], demonstrating that the neck linker can function like the Ncd C-terminus (Additional file 1 Figure S2). The reason for this is now apparent from the NcdT436S crystal structure, which shows that Ncd C-terminal residues form a structure in the same conformation as the neck linker - the kinesin-1 neck linker can replace the Ncd C-terminus to produce force, resulting in minus-end Ncd movement.

The structural and conformational similarities of NcdT436S to KIF1A-ATP, in the context of the kinetic data presented here, suggest that the orientation of the neck and C-terminus in chain B of the NcdT436S structure reflects a microtubule-bound, force-generating conformation of Ncd. That is, when ADP is bound, the neck would take on the orientation seen in chain A. Release of ADP would then cause the neck to reposition itself into the position seen in chain B and the C-terminus to dock, resulting in net movement towards the minus end of the microtubule. Remarkably, the same changes in the motor core of other kinesin motors, such as kinesin-1 and kinesin-3 (KIF1A), would trigger the docking of the neck linker onto the motor core, resulting in net movement in the plus end direction. It thus appears that plus- and minus-end directed motors have harnessed virtually identical conformational changes in the motor core and then, using distinct linkers, amplified these changes into movement in opposite directions.

### Ncd stalk rotation

The NcdT436S mutant has a single amino acid change in the conserved nucleotide-binding P loop that causes the motor to release ADP and hydrolyze ATP faster than wild type, but move more slowly along microtubules in gliding assays, uncoupling ATP hydrolysis from force generation. Unexpectedly, the NcdT436S crystal structure shows the stalk rotated ~70° relative to previous motor-ADP structures [2,3]; this was also observed in an earlier crystal structure of NcdN600K, a motor with a single amino acid change in the microtubule-binding helix α4 [13]. The previous motor-ADP structures have been interpreted to represent the Ncd pre-power stroke state and the stalk-rotated conformation, the post-power stroke state [13,15]. The NcdN600K mutation is also an uncoupling mutant like NcdT436S; it blocks microtubule-stimulated ATP hydrolysis by the motor and movement by the motor along microtubules [31]. Surprisingly, the crystal structure of the NcdT436S motor shows the same large rotation of the stalk and one head as the NcdN600K structure, even though the mutated residue, T436S, is removed from N600K, on the opposite side of the motor.

Stalk rotation in the two motors is correlated with ADP release - ADP release is faster than wild type by 10-fold in NcdT436S and by ~1.6-fold in NcdN600K [13]. ADP release could cause a conformational change in the motor that results in undocking of a head from its neck, triggering the stalk rotation, which is then stabilized by docking of the C-terminus onto the motor core. Residues of the Ncd C-terminus following K674 may fold back onto the motor core or neck helix to further stabilize the rotated stalk and interact with the microtubule, as proposed for KCBP [16], consistent with the finding that this region of the motor, which is highly positively charged, is required to bind microtubules to the glass surface in motility assays [30].

The finding that stalk rotation is correlated with ADP release contradicts the conclusion that the Ncd stalk rotates when the microtubule-bound motor binds ATP, based on laser trap assays or cryoEM reconstructions [11,14,15]. As noted above, Ncd stalk rotation requires the breaking of a series of salt bridges between the head and neck that stabilize the motor-ADP state and the formation of new salt bridges that stabilize the stalk-rotated conformation. ADP release could initiate stalk rotation, which may occur in steps corresponding to the breaking of salt bridges and formation of new ones, induced by conformational changes caused by motor interactions with the microtubule and ATP binding. This would produce the stalk-rotated conformation in which the head with the bound C-terminus is undocked from its neck, whereas the head that rotates with the stalk remains docked onto its neck.

### NcdT436S vs kinesin-1 T94S

The kinesin-1 T94S mutation corresponding to NcdT436S caused the motor to take 16-nm steps along microtubules under high load that consisted of rapid double 8-nm steps with a short dwell between steps, followed by a longer dwell [9]. The long dwell times may reflect the tendency of the forward head to release ADP while the motor remains bound to the microtubule, pausing between steps, and the short dwell times, accelerated release of the rear head due to interactions between the forward and rear heads. The NcdT436S mutant may have a similar tendency to pause between cycles of ATP hydrolysis. This, together with movement towards the minus end, could cause minus-end microtubule disassembly in ensemble gliding assays. The NcdT436S crystal structure shows the motor in a conformation that is thought to resemble a microtubule-bound form of the motor, even in the absence of microtubules. Opening the nucleotide-binding cleft probably destabilizes the bound ADP by altering water-mediated coordination of the ADP or Mg^+2 ^[32]; the motor then binds and hydrolyzes ATP, and releases ADP, repeating this cycle at a rate 2 to 3 times faster than wild type in the presence or absence of microtubules. The slower velocity of movement along microtubules means that ATP hydrolysis by the NcdT436S motor is not tightly coupled to force generation - the stalk does not rotate with each ATP hydrolysis.

Changing the invariant threonine to serine in the nucleotide-binding P loop of kinesin-1 or Ncd thus destabilizes the motor-ADP state, uncoupling force generation from nucleotide hydrolysis. The mutants reveal a force-sensitive phase of the kinesin-1 stepping cycle [9] and a conformation of Ncd that is thought to be force-producing. Mutants that decouple other steps of the nucleotide binding and hydrolysis cycle will be invaluable in addressing the basis of mechanochemical coupling and force production by the kinesin motors.

## Conclusions

A crystal structure of a kinesin-14 mutant in which the ADP release rate is much higher than wild-type Ncd shows the motor in a force-producing conformation with the stalk and one head rotated relative to the other head. The visible C-terminus residues in the unrotated head are docked onto the motor core in a conformation resembling the neck linker of kinesin-1. The C-terminus of minus-end Ncd thus appears to be involved in force production by the motor, performing a role similar to the neck linker of plus-end kinesin-1 and explaining the functional requirement for the Ncd C-terminus demonstrated in previous motility assays of chimeric Ncd-kinesin-1 motors. The new crystal structure provides new information about the role of the Ncd C-terminus in producing force - it implies that the plus- and minus-end kinesin motors have similar mechanisms, despite their structural differences. The state in which the stalk rotates is currently controversial, but the results presented here indicate that stalk rotation is correlated with ADP release, rather than ATP binding, as proposed by others.

## Methods

### Protein expression and purification

Plasmids for NcdT436S expression were constructed using conventional methods. Protein for crystallization (MGSM-H293-K700; 93,692 Da) or biochemical and motility assays (GST-SDLVPRGSPV-K210-K700; 164,642 Da) was expressed in *BL21(DE3)pLysS *or *Rosetta2(DE3)pLysS *(Novagen) cells. Protein purification was by chromatography on SP-Sepharose FF, followed by FPLC on MonoQ and/or Superose 12 [33].

### Transient and steady-state kinetic assays

Single-turnover ADP release assays were performed using FPLC-purified GST/NcdT436S or the corresponding wild-type GST/Ncd (GST/MC1) [34] protein (0.4-0.5 μM), as described [9,31]. Protein was incubated with mant-ATP (2'(3')-O-(N-methyl-anthraniloyl)-adenine 5'-triphosphate) in HEM100 (10 mM HEPES pH 7.2, 1 mM EGTA, 1 mM MgCl_2_, 100 mM NaCl) at 22°C for ~10 min, then on ice >1 hr, and assayed (λ_ex _= 356 nm, λ_εμ _= 446 nm) for 600 s; 0.5 mM MgATP was added and assays were continued up to 1600 s. Data points were fit to y = m3 + m2*e^-m1*t^, where y = fluorescence, m3 = fluorescence at t = ∞, m2 = total fluorescence loss, m1 = k_off _and t = time (s). Assays with microtubules were performed with 0.2-0.25 μM motor in HEM50 by adding 0.94 μM polymerized tubulin at 60 s and 0.5 mM MgATP at 600 s or 650 s; data were analyzed in the same way. Steady-state ATPase assays were performed using 0.5 μM FPLC-purified protein, 1 mM MgATP and 0-2.5 μM microtubules using a coupled-enzyme assay, as described [31]. Data were fit to the Michaelis-Menton equation to estimate the basal ATPase rate, microtubule-stimulated k_cat _and K_m,MTs_.

### Motility assays

Lysates were prepared from bacterial cells expressing GST/NcdT436S or wild-type GST/Ncd and microtubule gliding assays were performed, as described [35]. Microtubules were tracked from videotape to determine motor velocities.

### Crystallization

Following purification, protein was concentrated to 4.5 mg/ml and incubated on ice for 120 minutes in the presence of 4 mM MgCl_2 _and 4 mM ATP. At the end of the incubation period, DTT was added to the protein mixture to a final concentration of 7 mM. Hanging drops were set up at room temperature using a 1:1 ratio of protein to a reservoir solution containing 11.5% PEG 8K, 25 mM sodium-phosphate buffer pH 6.26, 3.5 mM DTT, 10 mM MgCl_2_, and 0.5 M NaCl. Crystals were frozen using 40% ethylene glycol as a cryoprotectant and stored in liquid nitrogen for data collection.

### X-ray data collection and structure solution

A dataset was collected at National Synchrotron Light Source beamline X6A at Brookhaven National Laboratory. Diffraction data were indexed with XDS [36], solved using CNS v1.2 [37,38] by molecular replacement using PDB 1N6M[13] as a search model, and refined with CNS v1.2. The model was built using WinCoot [39,40]. The position of all residues was confirmed using a composite omit map generated with CNS v1.2. A Ramachandran plot generated with RAMPAGE [41] shows 100% of residues in the favored or allowed regions and no residues in outlier regions. X-ray data collection and refinement statistics are given in Table 2. Residues missing in the refined model because of the absence of visible electron density were chain A residues 386-391, 539-550 and 591-597, and chain B residues 467-472, 495-516, 540-548, 567-569 and 588-599.

## Authors' contributions

EH crystallized NcdT436S and solved the structure, CEB purified the mutant and wild-type proteins and performed the biochemical assays, FJK supervised the structural solution and analysis, made figures and helped write the manuscript, SAE constructed plasmids, supervised protein purification and data analysis, made figures and wrote the manuscript with FJK All authors read and approved the final manuscript.

## Supplementary Material

Additional file 1 **Supplementary Figures**. 1) Figure S1 shows that there is a difference in the ADP density in the two heads of the NcdT436S model consistent with the occurrence of stalk rotation with ADP release; it is reduced in the head of chain B that is positioned to bind to the microtubule compared to the head of chain A. 2) Figure S2 shows the kinesin-1 neck linker and Ncd neck mimic, together with a diagram of their positions in the two motors and a diagram of an Ncd motor with the neck mimic replaced by the kinesin-1 neck linker that showed minus-end motility. Click here for file
