# Supplementary material for: A kinesin motor in a force-producing conformation
Source: BMC Struct Biol. 2010 Jul 5;10:19. doi: 10.1186/1472-6807-10-19 (PMC2906495; doi:10.1186/1472-6807-10-19)
Supplement: Additional file 1 — Supplementary Figures. 1) Figure S1 shows that there is a difference in the ADP density in the two heads of the NcdT436S model consistent with the occurrence of stalk rotation with ADP release; it is reduced in the head of chain B that is positioned to bind to the microtubule compared to the head of chain A. 2) Figure S2 shows the kinesin-1 neck linker and Ncd neck mimic, together with a diagram of their positions in the two motors and a diagram of an Ncd motor with the neck mimic replaced by the kinesin-1 neck linker that showed minus-end motility. [file 1472-6807-10-19-S1.DOC]

A Kinesin Motor in a force-producing conformation

**Elisabeth Heuston^1^, C. Eric Bronner^2^, F. Jon Kull^1^, Sharyn A. Endow^2^**

^1^Department of Chemistry, Dartmouth College, Hanover, NH 03755 USA

^2^Department of Cell Biology, Duke University Medical Center, Durham, NC 27710 USA

# Additional Figures


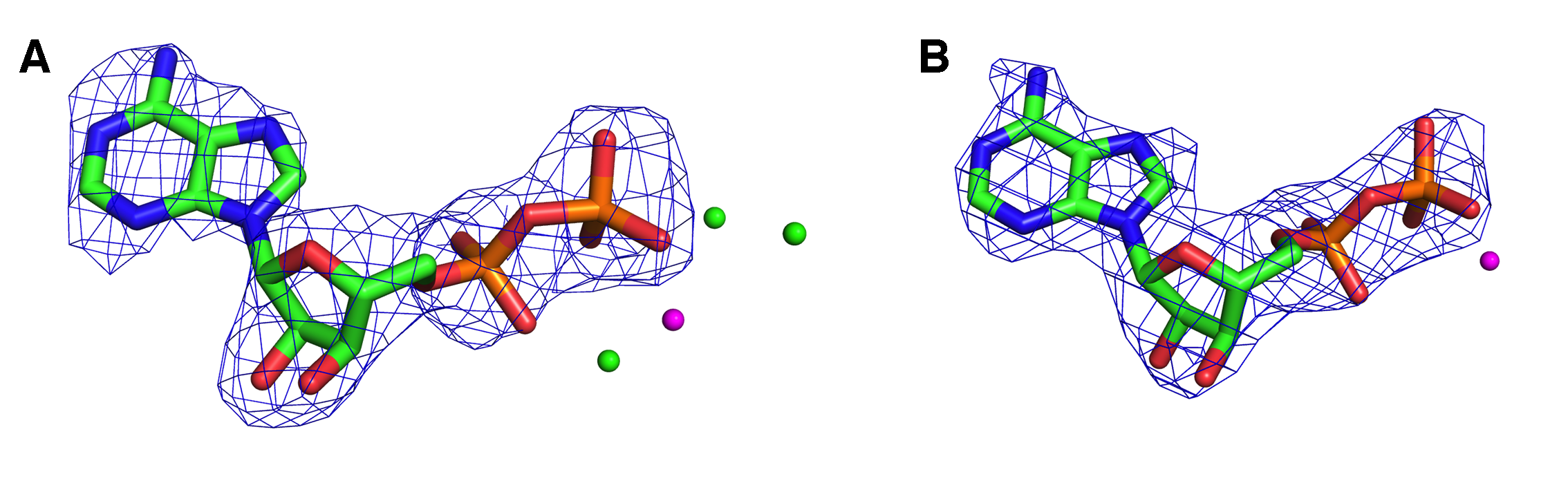


**Figure S1 ADP bound to NcdT436S heads.** *A,* ADP density in chain A head. Mg^+2^, magenta sphere; water, green spheres. 2F_o_-F_c_ electron density map contoured at 1σ. For clarity, the density for the Mg^+2^ and water molecules is not shown. *B,* Density corresponding to the bound ADP in chain B head is reduced. Electron density for the Mg^+2^ is present, but density for the water molecules visible in the chain A head is absent.


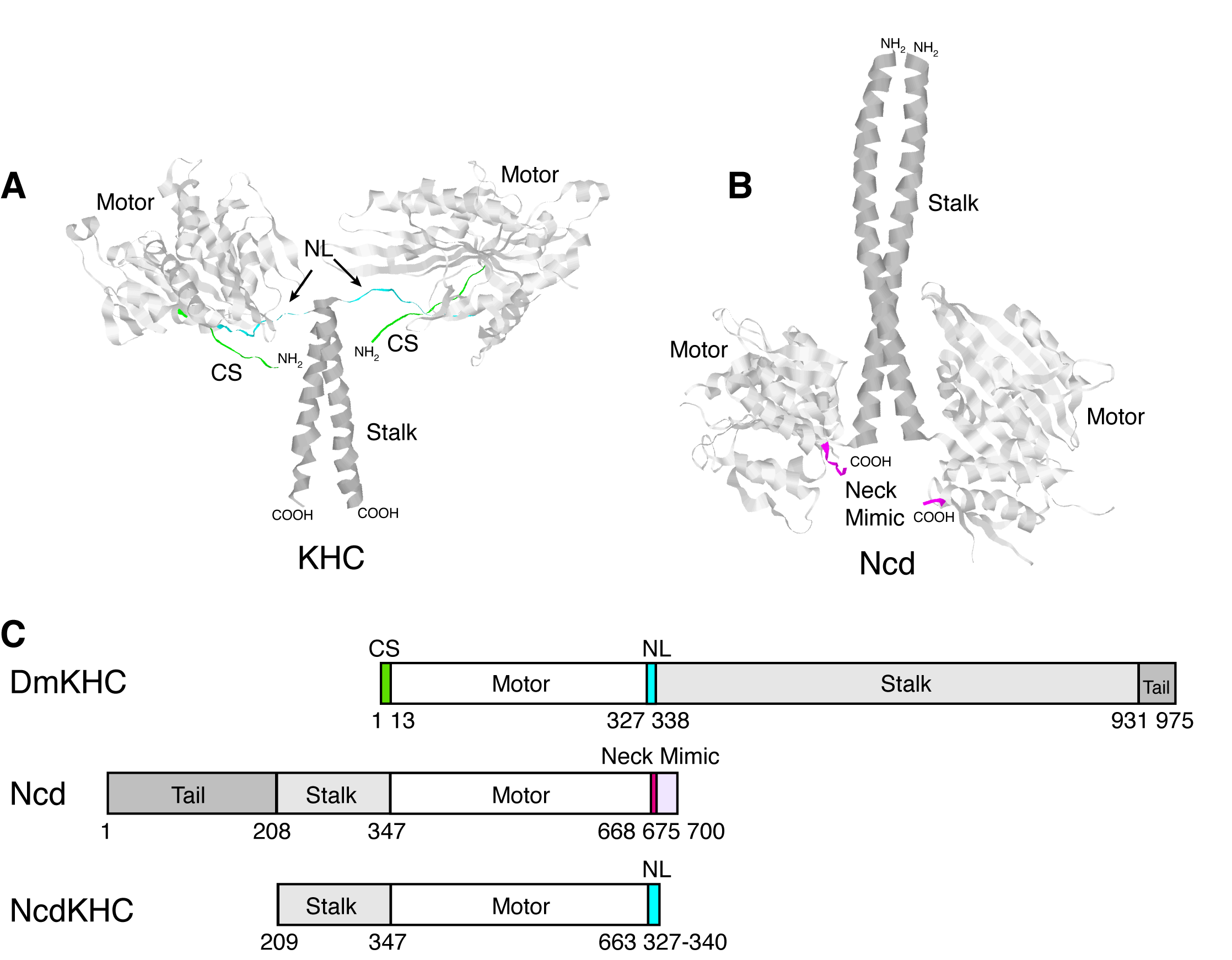


**Figure S2 Ncd neck mimic and kinesin-1 neck linker.** *A,* Kinesin heavy chain (KHC) heads attach to the stalk (gray) via the neck linker (NL, cyan) (PDB 3KIN). The N-terminal cover strand (CS, green) has been hypothesized to interact with the neck linker in the ATP state, forming the cover-neck bundle [1, 2]. *B,* Ncd heads attach to the stalk without a neck linker. Residues corresponding to the cover strand dock onto the chain B head (left) in a structure resembling the neck linker – the neck mimic (magenta). *C,* Diagram showing DmKHC (*Drosophila* *melanogaster* KHC) cover strand and neck linker (top), and Ncd neck mimic (middle). The Ncd neck mimic is required for minus-end directed motility of chimeric NcdKHC motors; replacing the Ncd neck mimic with the DmKHC neck linker resulted in minus-end motility of wild-type Ncd (bottom) [3]. Abbreviations and colors as in (*A*) and (*B*).

**References**

1. Hwang W, Lang MJ, Karplus M: **Force generation in kinesin hinges on cover-neck bundle formation**. *Structure* 2008, **16**:62-71.

2. Khalil AS, Appleyard DC, Labno AK, Georges A, Karplus M, Belcher AM, Hwang W, Lang MJ: **Kinesin's cover-neck bundle folds forward to generate force**. *Proc Natl Acad Sci USA* 2008, **105**:19247-19252.

3. Endow SA, Waligora KW: **Determinants of kinesin motor polarity**. *Science* 1998, **281**:1200-1202.
